# Diabetes Myonecrosis: A Debilitating Complication in an Indigenous Young Woman With Long Standing Type 1 Diabetes Mellitus

**DOI:** 10.1155/2024/8839798

**Published:** 2024-09-27

**Authors:** Jinwen He, Liyan Wang, Thomas Robertson, Swetha Rangaswamaiah, Usman H. Malabu

**Affiliations:** ^1^ Department of Diabetes and Endocrinology Townsville University Hospital, Townsville, Australia; ^2^ Department of Anatomical Pathology Royal Brisbane and Women's Hospital, Herston, Australia; ^3^ College of Medicine and Dentistry James Cook University, Townsville, Australia

## Abstract

A 24-year-old Indigenous Australian female with long-standing, poorly controlled type 1 diabetes mellitus (T1DM) presented with 3 months' history of unilateral thigh swelling and pain. Her laboratory investigations showed evidence of a persistent inflammatory state with normal creatine kinase. Infectious and autoimmune investigations were negative. Imaging demonstrated evidence of muscular oedema and atrophy. Muscular pain and swelling have a broad list of differential diagnoses. This case highlights a rare but potentially debilitating complication of diabetes mellitus—diabetic myonecrosis with its challenges in reaching a definitive diagnosis due to non-specific symptomology and laboratory findings. However, it is an important differential of leg pain and swelling to consider, particularly in those with long-standing diabetes and pre-existing microvascular complications. Glycaemic control is paramount in preventing this potentially severe diabetic complication.

## 1. Introduction

Type 1 diabetes mellitus (T1DM) is a relatively uncommon condition in the Australian Indigenous population, with an incidence rate of 7 per 100,000 persons compared to 10 per 100,000 persons in non-Indigenous counterparts [1]. A retrospective analysis of Aboriginal people in remote communities of the Northern Territory in Australia by Hare et al. [2] found T1DM making up only less than 0.2% of all diabetes. In comparison, the incidence of type 2 diabetes mellitus (T2DM) is about four times higher among Indigenous Australians compared to non-Indigenous counterparts [1]. Additionally, the rates of diabetes complications and associated hospitalisations are also higher in Indigenous Australians [1]. Management of diabetes and its complications in Indigenous Australians often pose challenges due to significant social and cultural barriers as well as geographical isolation.

We present a case of diabetic myonecrosis in an Indigenous Australian female with T1DM. This condition is typically described as spontaneous atraumatic ischaemic necrosis of skeletal muscle and is a rare microvascular complication associated with poor glycaemic control. Patients typically present with painful muscular swelling that evolves over days or weeks. Diagnosis is often delayed due to non-specific symptomology and a broad list of differential diagnoses, including deep vein thrombosis, pyomyositis, muscle abscess, necrotising fasciitis, muscle neoplasm, haematoma, inflammatory myositis, diabetic amyotrophy, and more [3–5].

## 2. Case Presentation

A 24-year-old Indigenous Australian female from a remote community presented with a 3-month history of left thigh swelling and pain with limitation of movement. She has a long-standing history of poorly controlled T1DM diagnosed at the age of 14 years. Her HbA1c was 12.4% (114 mmol/mol) on admission, which had historically been as high as 20.9% (204.9 mmol/mol). She had multiple diabetic microvascular complications, including bilateral proliferative retinopathy, nephrotic-range proteinuria, peripheral neuropathy, and autonomic neuropathy with associated urinary retention. Her family history includes T2DM in her brother, as well as diabetes of unclear type with associated end stage renal failure in her mother.

Other past medical history includes acute rheumatic fever, syphilis, and recurrent urinary tract infections (UTIs) associated with urinary retention. She has an active smoking history of up to 20 cigarettes per day. Past surgical history includes open reduction internal fixation of right neck of femur following fracture from a motor vehicle accident. Her only prescribed medication was premixed insulin comprised of degludec and aspart at a dosage of 14 units twice daily with meals.

Clinical examination revealed a pale, cachectic, and undernourished young female weighing 32.5 kg with a BMI of 12.9 kg/m2. She had a sinus tachycardia of 120 bpm. Other vital signs were within normal limits. She had significant left thigh swelling that was tender on palpitation. However, there was no erythema, warmth to touch, or skin colour changes. Her peripheral pedal pulses were palpable bilaterally. Neurological examination was limited by pain but revealed peripheral neuropathy in a stocking distribution, as well as reduced power in the proximal left lower limb. Her visual acuity was reduced to counting fingers bilaterally. Fundal examination revealed severe bilateral proliferative diabetic retinopathy with macular oedema.

Investigations showed normocytic anaemia with haemoglobin (Hb) of 90 g/L (9 g/dL) and elevated inflammatory markers, namely, platelets (649 × 10^9^/L), white cell count (20.3 × 10^9^/L), neutrophil (17.43 × 10^9^/L), erythrocyte sedimentation rate (120 mm/h), c-reactive protein (185 mg/L and 18.5 mg/dL), and ferritin (1290 µg/L and 1290 ng/mL) as shown in Table 1. She had impaired renal function with reduced creatinine clearance at 50 mL/min. Creatine kinase (CK) and liver function tests were normal. She was biochemically euthyroid, and morning cortisol was robust at 528 nmol/L (19.14 µg/dL). A nutritional panel showed severe hypoalbuminaemia (<15 g/L), low vitamin D (10.3 nmol/L), and low vitamin C (12 µmol/L). Coeliac serology was undetectable.

Urine microscopy and culture showed evidence of a UTI, and albumin/creatinine ratio was raised at 840 g/mol consistent with nephrotic-range proteinuria. Renal tract ultrasound showed cystitis, and renal biopsy confirmed Class III diabetic nephropathy with 30% global glomerulosclerosis and 60% interstitial fibrosis and tubular atrophy.

**Figure 1:**
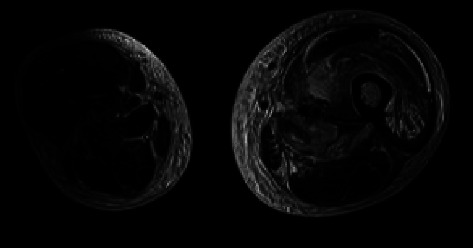
T1-weighted MRI (axial) showing muscular oedema of left thigh—affecting rectus femoris, vastus medialis, vastus lateralis, and adductor tendons including the adductor longus, adductor brevis, adductor magnus, adductor gracilis, and pectineus muscles.

Computed tomography (CT) scan of her pelvis and leg showed ill-defined diffuse low attenuation within the left mid-thigh muscle measuring 36 × 34 × 150 mm. There were no pelvic masses, collections, or any bony abnormalities. A Doppler ultrasound excluded a deep vein thrombosis. Subsequent magnetic resonance imaging (MRI) showed significant muscle oedema of the left anterior thigh in comparison to the right side, affecting rectus femoris, vastus medialis, vastus lateralis, and adductor muscles (Figure 1). The muscles were atrophic with fatty and fluid replacement, and the subcutaneous tissue was oedematous. The posterior aspect was not affected.

She remained in a persistent inflammatory state despite appropriate treatment of infections including UTI and hospital acquired pneumonia (HAP). A CT pulmonary angiogram was negative for pulmonary embolism, and a transthoracic echocardiogram showed normal left and right ventricular size and function.

**Figure 2:**
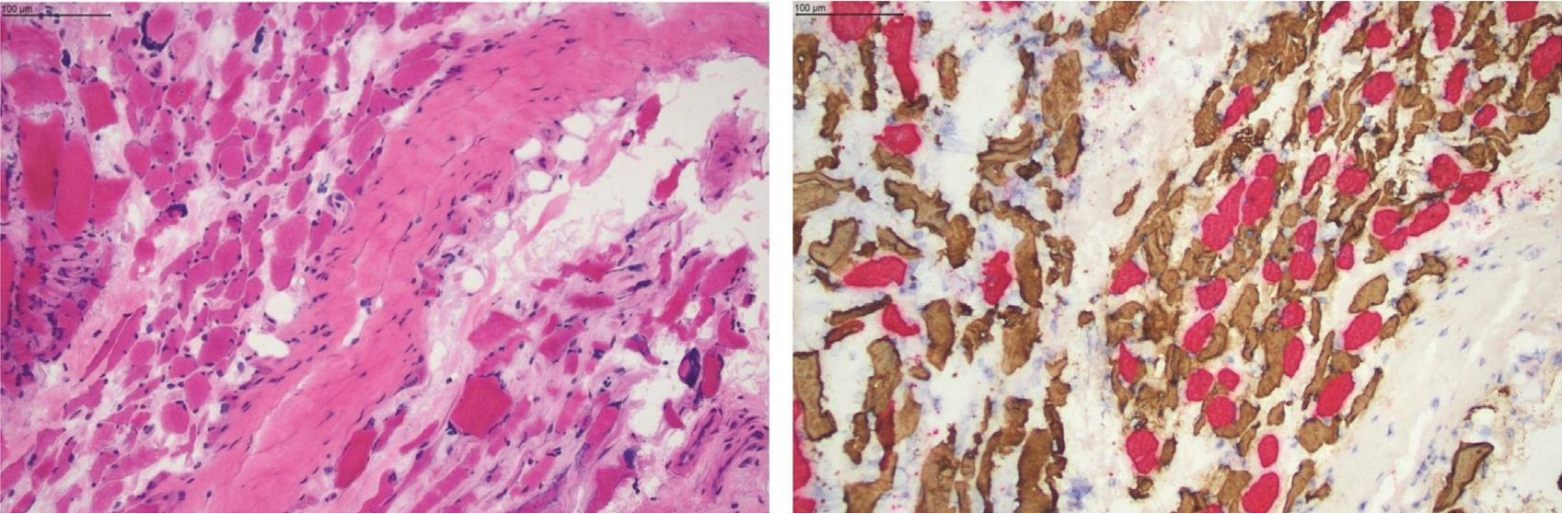
Muscle biopsy showing evidence of severe atrophy, predominantly involving the fast fibres. Left panel with haematoxylin and eosin staining showing increased interstitial fibrosis (pale pink between dark pink muscle). Right panel with fast and slow myosin double immunohistochemistry stain (red is slow myosin, and brown is fast myosin).

The differential diagnoses considered at this stage included diabetic myonecrosis, nutritional myopathy, and inflammatory myositis. Infectious aetiologies were also screened for, with unremarkable results (including 18-fluorodeoxyglucose positron emission tomography). Subsequent autoimmune testing including a myositis autoantibody panel was negative. A muscle biopsy was taken about 3 months after onset of symptoms. This showed significant autolytic changes and widespread muscle atrophy, with partial replacement of muscle mass by interstitial fibrosis and adipose tissue (Figure 2). There was no evidence of inflammation and no active muscle fibre necrosis. The biopsy findings were interpreted as loss of muscle mass through mixed aetiologies, including previous necrosis and ischaemic atrophy, immobility, and denervation. Microscopy and culture of muscle biopsy specimen showed no bacterial growth or acid-fast bacilli. Nerve conduction studies (NCS) and electromyography (EMG) were attempted but unsuccessful due to the patient's intolerance.

Management was supportive with optimisation of glycaemic control and nutrition and pain management along with treatment of underlying infections. Given her persistent inflammatory state, she was treated empirically with intravenous amoxycillin/clavulanate for 1 week whilst awaiting the result of her muscle biopsy. Her insulin regimen was changed from premixed to basal-bolus insulin and was closely monitored on a flash glucose monitoring system. Nutrition was optimised with a high protein diet, additional nutritional supplement drinks, vitamin C and D, thiamine, and multivitamin supplements.

Pain and mobility improved throughout her hospital admission, and inflammatory markers were normalised. She was transferred back to her local hospital for ongoing rehabilitation prior to discharge. Follow-up HbA1c had improved down to 8.7% (71.6 mmol/mol), and nutritional status improved with weight gain from 32.5 kg to 37.5 kg.

## 3. Discussion

This case posed a diagnostic challenge. The main differential diagnoses were diabetic myonecrosis, diabetic amyotrophy, and nutritional myopathy. Subsequent investigations ruled out infectious myositis and autoimmune myositis. The clinical presentation and progress, as well as imaging features of our case, were in keeping with diabetic myonecrosis, despite having a negative biopsy for muscular necrosis. The potential reasons for this are likely due to the delayed biopsy by 3 months following onset of symptoms, as well as the suboptimal quality of the muscle specimen (due to the preceding collection of multiple microbiology specimens). Our patient had multiple risk factors for this condition, including long-standing poor glycaemic control and multiple microvascular complications. As far as we know, this is the first case report of the rare complication of diabetes in an Indigenous Australian with T1DM.

Diabetic myonecrosis is the spontaneous atraumatic ischaemic necrosis of skeletal muscle in patients with poorly controlled diabetes, which presents with painful muscular swelling that evolves over days or weeks. It was first reported in 1965 by Angervall and Stener, who termed it ‘tumouriform focal muscle degeneration' [6]. Since then, it has also been reported in the literature as diabetic muscle infarction, aseptic myonecrosis, and ischaemic myonecrosis. The pathophysiology is unclear but postulated to be secondary to atherosclerosis, diabetic microangiopathy, vasculitis with thrombosis, and ischaemia–reperfusion injury. These processes subsequently lead to an inflammatory cascade and resultant muscular ischaemia and necrosis [4, 5].

A systematic review by Horton et al. in 2015 described 126 published cases of diabetic muscle infarction. Of the 108 cases with reported diabetes type, 50% had T2DM, and 41.7% had T1DM. These patients had poorly controlled diabetes with a mean HbA1c of 9.3% (78 mmol/mol) and 65.8% having at least two microvascular complications. In this review, most patients (89%) were afebrile, and more than half (57.5%) had normal white cell count. However, the majority of patients had elevated CRP (90%) and ESR (83.3%). Only 31.6% of patients had raised CK. Commonly affected sites were thighs (71.2%), followed by calves (15.3%) and upper arm (5.4%) [4].

MRI is the preferred imaging modality. Characteristic MRI features include a hyperintense signal on T2-weighted images (due to increased water content from oedema) and an isointense to hypointense signal on T1-weighted images from the affected muscle, with associated perifascial, perimuscular, and/or subcutaneous oedema [4, 7]. Post-gadolinium, there may be diffuse heterogeneous enhancement or rim enhancement [8–10]. These imaging features are not specific and can be found in other causes of myositis including autoimmune and infectious myositis [10]. Ultrasound features include a well-marginated, hypoechoic, and intramuscular lesion with internal linear echogenic structures [7].

Muscle biopsy is not essential for diagnosis and should be reserved for cases where the presentation is atypical [4, 7, 8]. Biopsy was reported in 63 out of 119 cases in the recent 2015 systematic review of cases, with mean time to symptom resolution being longer when biopsy was performed (60.8 days), compared to when it was avoided (29.5 days) [4]. Biopsy usually shows areas of muscle necrosis and oedema, with later findings of fibrotic tissue, muscle fibre regeneration, and mononuclear cell infiltration [4, 5, 7].

Management of diabetic myonecrosis is supportive, as in our patient, with tight glycaemic control, bed rest, and nonsteroidal anti-inflammatory drugs (NSAID) being the mainstays of treatment [4, 7]. Physiotherapy is controversial with some studies showing that physiotherapy in the acute phase may prolong recovery [4]. Patients who received surgical treatment also had prolonged recovery, although it is not clear whether the surgical group had more severe disease with complications and hence indications for surgery [4].

On the contrary, diabetic amyotrophy is a subacute and progressive condition, with afflicted patients presenting with severe unilateral proximal leg pain, followed by weakness, wasting, and weight loss. It is also known as diabetic lumbosacral plexopathy and Bruns–Garland Syndrome. Unlike in diabetic myonecrosis, it can affect patients with good glycaemic control [11] and seems to be more common in patients with T2DM [12]. Pain typically involves the thigh, hip, or back and is unilateral in most cases, followed by atrophy and weakness of the affected muscles within a few weeks. Significant weight loss at onset is common, and autonomic dysfunction affects up to 20% of patients [12]. NCS reveal an amplitude drop of the nerves of the affected region suggestive of axonal neuropathy. EMG of the involved muscles shows active denervation with or without reinnervation, indicative of a lumbosacral plexopathy [11].

A nutritional myopathy was also considered due to the patient's low BMI and micronutrient deficiencies. However, it was thought to be less likely in light of clinical presentation and imaging features. Vitamin C deficiency or scurvy can cause musculoskeletal pain due to intramuscular haemorrhage. Case reports of vitamin C deficiency causing severe myopathy and weakness have been reported in the literature [13]. The clinical features and investigation findings in our patient, however, were not in keeping with this, given the absence of haemorrhage, absence of other features of scurvy, and pain being the more prominent symptom rather than weakness. Severe vitamin D deficiency has also been reported to cause proximal myopathy with muscular pain and weakness [14]. However, this is typically more widespread and bilateral rather than unilateral, as with our patient.

This case report showcases the difficulty in diagnosing diabetic myonecrosis and the often numerous investigations that patients undergo prior to diagnosis. Diabetes complication screening is an important aspect of routine diabetes care, and rare complications such as diabetic myonecrosis should be considered on the list of differentials. Glycaemic control is paramount in the prevention of this potentially debilitating complication. However, this, along with extensive investigations, can be difficult to achieve in geographically isolated and resource-limited communities.

## 4. Conclusion

Diabetic myonecrosis is a rare complication of long-standing and poorly controlled diabetes mellitus that can cause significant morbidity and reduce quality of life. Diagnosis is based on clinical and imaging features and, therefore, requires a high index of suspicion in patients with long-standing diabetes with microvascular complications, given the similarity of the presentation to multiple other conditions.

## Figures and Tables

**Table 1:** Laboratory investigation results.

Laboratory parameter	Result	Reference ranges
Hb	90 g/L (9 g/dL)	115–160 g/L
MCV	85 fl	80–100 fL
MCH	26.6 pg	27.5–33 pg
Hct	0.29 (29%)	0.33–0.47
Plat	996 × 10^9^/L	140–400 × 10^9^/L
WCC	16 × 10^9^/L	4–11 × 10^9^/L
Neut	11.97 × 10^9^/L	2–8 × 10^9^/L
Lymph	2.44 × 10^9^/L	1–4 × 10^9^/L
Reticulocytes	46 × 10^9^/L	10–100 × 10^9^/L
Na	134 mmol/L (134 mEq/L)	135–145 mmol/L
K	4.7 mmol/L (4.7 mEq/L)	3.5–5.2 mmol/L
Creatinine	81 µmol/L (0.92 mg/dL)	45–90 µmol/L
eGFR	>90 mL/min/1.73 m^2^	>90 mL/min/1.73 m^2^
Albumin	<15 g/L (<1.5 g/dL)	32–45 g/L
CK	66 U/L (1.1 ukat/L)	34–145 U/L
CRP	185 mg/L (18.5 mg/dL)	<5 mg/L
ESR	120 mm/h	<12 mm/h
Ferritin	1290 µg/L (1290 ng/mL)	5–150 µg/L
TSH	1.45 mU/L (1.45 *μ*IU/mL)	0.3–4.5 mU/L
fT4	13.9 pmol/L (1.08 ng/dL)	11.5–22.1 pmol/L
ACTH	35 ng/L (35 pg/mL)	10–50 ng/L
Cortisol	528 nmol/L (19.14 µg/dL)	140–640 nmol/L
Rheumatoid factor	<20 IU/mL	<20 IU/mL
Anti-CCP	0 U/mL	<6 U/mL
Anti-dsDNA	5 IU/mL	<7 IU/mL
C3	2.29 g/L (229 mg/dL)	0.9–1.8 g/L
C4	0.69 g/L (69 mg/dL)	0.1–0.4 g/L
Lupus anticoagulant	Negative	—
ANA	Negative	—
ANCA	Negative	—
Anti-GBM	<3 CU	<20 CU
ACE (mass)	81 µg/L	37–211 µg/L
Myositis antibodies (Mi-2, Jo-1, PmScl, SRP, Ku, Pl-7, Pl-12, EJ, OJ, NXP2, SAE)	Negative	—
Anti-TTG IgA	2 CU	<20 CU
Vitamin B12	293 pmol/L (397 pg/mL)	133–680 pmol/L
Folate	16.7 nmol/L (7.36 ng/mL)	>7 nmol/L
Vitamin A	2.3 µmol/L (65.9 µg/dL)	1.1–2.8 µmol/L
Vitamin C	13 µmol/L (0.23 mg/dL)	20–120 µmol/L
Vitamin D	10.3 nmol/L (4.12 ng/mL)	50–150 nmol/L
1,25-Vitamin D	<12 pmol/L (<4.6 pg/mL)	48–190 pmol/L
Vitamin E	43 µmol/L (1.85 mg/dL)	11–45 µmol/L
Vitamin B6	90 nmol/L (22.2 ng/mL)	40–170 nmol/L
Thiamine diphosphate (whole blood)	3.29 nmol/g Hb	0.9–1.95 nmol/g Hb
Copper	36 µmol/L (229.3 µg/dL)	11–24 µmol/L
Zinc	8 µmol/L (52.3 µg/dL)	8–18 µmol/L
Selenium	0.8 µmol/L (6.32 µg/dL)	0.7–1.4 µmol/L

Abbreviations: ACE, angiotensin-converting enzyme; ACTH, adrenocorticotrophic hormone; ANA, antinuclear antibody; ANCA, anti-neutrophil cytoplasma antibody; Anti-CCP, anti-cyclic citrinullated peptide; Anti-dsDNA, anti-double-stranded deoxyribonucleic acid; Anti-GBM, anti-glomerular basement membrane; Anti-TTG, anti-tissue transglutaminase; CK, creatine kinase; CRP, c-reactive protein; eGFR, estimated glomerular filtration rate; ESR, erythrocyte sedimentation rate; fT4, free thyroxine; Hb, haemoglobin; Hct, haematocrit; K, potassium; Lymph, lymphocytes; MCH, mean corpuscular haemoglobin; MCV, mean corpuscular volume; Na, sodium; Neut, neutrophils; Plat, platelets; TSH, thyroid-stimulating hormone; WCC, white cell count.

## Data Availability

Data sharing is not applicable to this article as no new data were created or analysed in this study.
